# Informativeness across Interpreting Types: Implications for Language Shifts under Cognitive Load

**DOI:** 10.3390/e25020243

**Published:** 2023-01-28

**Authors:** Yumeng Lin, Junying Liang

**Affiliations:** Department of Linguistics, Zhejiang University, 866 Yuhangtang Road, Hangzhou 310058, China

**Keywords:** interpreting types, entropy, information content, distribution patterns, cognitive load

## Abstract

Previous quantitative studies discussing interpreting types have focused on various features of linguistic forms in outputs. However, none of them has examined their informativeness. Entropy, as a measure of the average information content and the uniformity of the probability distribution of language units, has been applied to quantitative linguistic research on different types of language texts. In the present study, entropy and repeat rate were used to investigate the difference of overall informativeness and concentration of output texts between simultaneous interpreting and consecutive interpreting. We intend to figure out the frequency distribution patterns of word and word category in two types of interpreting texts. Analyses of linear mixed-effects models showed that entropy and repeat rate can distinguish the informativeness of consecutive and simultaneous interpreting outputs, and consecutive interpreting outputs entail a higher word entropy value and a lower word repeat rate than simultaneous interpreting outputs. We propose that consecutive interpreting is a cognitive process which reaches an equilibrium between production economy for interpreters and comprehension sufficiency for listeners, especially in the case where input speeches are more complex. Our findings also shed lights on the selection of interpreting types in application scenarios. The current research is the first of its kind in examining informativeness across interpreting types, demonstrating a dynamic adaptation of language users to extreme cognitive load.

## 1. Introduction

Interpreting is a special case of bilingual/multilingual language use which involves more frequent code switching and greater inhibitory demands than language use in other settings [1]. The intricacies of interpreting lie in the extreme cognitive efforts required for accommodating continuous speech input [2], temporary storage, reformulation into the target language, and multimodal processes consisting in concurrent encoding and decoding [2,3,4,5,6,7,8,9]. Under such intense pressure, interpreters work close to saturation most of the time [10]. 

Although extremely high cognitive pressure exists across all types of interpreting, the most common conceptual distinction is made in terms of the temporal relationship between the target speech and the source speech, which yields consecutive interpreting (CI) and simultaneous interpreting (SI) [11]. SI is ‘the mode of interpreting in which the interpreter renders the speech as it is being delivered by a speaker into another language, with a minimal time lag of a few seconds’ [11], whereas in CI, interpreters await his or her turn while memorizing the source information with the help of the notes to subsequently reproduce it into the target language. 

### 1.1. Distinctive Processes in CI and SI

Interpreting models and theories have been proposed to conceptualize distinct interpreting processes in CI and SI. Gile [10] pointed out that SI can be modeled as a one-step process requiring several simultaneous efforts: listening and analysis, short-term memory, production, and coordination. By contrast, CI is modeled as two separate stages (that is, the comprehension phase is followed by the reformulation phase where the target speech is produced by reconstructing the source message from memory). The discrepancy in processing stages of SI and CI indicates that SI interpreters may retain the linguistic form of the source message to a larger extent than CI, which has been substantiated by an array of theoretical and quantitative studies. As claimed by Shlesinger [12], SI is processed in a linear manner and is strongly constrained by time. Hence, it is impossible for interpreters to process long chunks of message by formulating the meaning of many short units in the source speech all of the time. Such a feature is in line with the findings of Bacigalupe [13], in which the data of the interpreting production suggested that the target speech in SI is largely based on the external shape of the source speech and perhaps the meaning construction of the original utterances is not even a priority. By contrast, in CI, the phase of speech comprehension and production are separated in time. Interpreters can take down notes and reformulate the information into ‘a succession of natural-sounding target language sentences’ [10]. Thus, at the reformulation phase, CI interpreters are more self-paced with fewer syntactic constraints, giving priority to conveying the message of the source speech with more flexible sentence structures instead of word-for-word translation.

However, other than theories and models highlighting the distinct processes between CI and SI, there have been few efforts to compare the differences between SI and CI output directly. Among the few is Gile [14]’s attempt to compare the accuracy of SI and CI renderings in terms of faithfulness and overall fidelity. It was found that CI was superior in interpreting segments with incomplete sentences and that SI was superior as regards digressions and unimportant modifiers. With respect to overall accuracy, SI was clearly superior to CI. Conversely, Russel [15] found the opposite results in legal contexts, claiming that CI demonstrated a greater degree of accuracy than SI in terms of the number of interpreting errors. Although these studies offer valuable insights into the differences between SI and CI renderings by conducting experiments, diverse approaches such as quantitative methods may complement their findings in holistic terms and yield meaningful results. In the seminal research quantifying interpreting types [16], the index ‘dependency distance’ (the number of words intervening between two syntactically related words) was employed to quantify and account for syntactic difficulty and cognitive demands in SI and CI. The comparison of dependency distance of interpreting output texts in different interpreting modes showed that CI texts yield the smallest dependency distance other than those of other interpreting types. This result could be attributed to different cognitive demand between SI and CI. Another product-oriented study [17] compared SI and CI from a lexical perspective by examining lexical simplification parameters, and the results showed that the output of CI demonstrates the most simplified lexical pattern, further corroborating the prior findings. These results converge to suggest that heavier cognitive demands may be required in CI than other interpreting modes. Moreover, the fact that SI demonstrated larger dependency distance and less simplified lexical patterns indicated that, under severe cognitive pressure, SI interpreters are prone to retain both the syntactical and lexical linguistic form of the source text to a larger extent than CI interpreters. Apart from lexical and syntactic features, Liang et al. [18] probed into the sequence-related features which can visualize the local distribution of function words. By examining the distribution, length and the position-dependent properties of a language sequential unit, a frequency motif, in SI and CI, the authors manifested that the features of frequency motifs can distinguish SI and CI outputs and mirror different operational mechanisms between the two interpreting modes. To further tap into potential lexical-category-related features in interpreting texts, a recent study [4] compared the index of ‘activity’ (a normalized ratio between verb and adjective occurrences in the text) in the output texts of CI and SI. Again, a significant discrepancy between SI and CI was found after controlling for the confounding variables.

Previous studies discussing interpreting types focus on various features of linguistic forms and shed light on varied processing mechanisms and constraints underlying different interpreting modes. However, on the one hand, few studies investigate the frequency distribution patterns of language units across different types of interpreting texts. On the other hand, converging evidence manifests that the production of SI is highly constrained by the input, and hence retains the linguistic form of the inputs to a larger extent than CI. Given that the nature of interpreting lies in the faithful transmission of information from the source language to the target language, the coping mechanisms in CI and SI promote our interest in further investigating the relationship between information content and interpreting types. According to Shannon [19], any channel giving access to transmit information has a finite transmission capacity, beyond which information loss occurs. As is postulated in the Tightrope Hypothesis, interpreting requires types of attention-sharing and overloading of working memory [20], which can be close to the limits of cognitive processing capabilities. When the total cognitive load exceeds the interpreter’s available processing capacity, information overflow occurs [10]. Due to varied cognitive pressures in CI and SI, it is highly probable that the information processing mechanisms of CI and SI differ, and the information content or the distribution of language units in their output texts may show distinct patterns.

### 1.2. Entropic Measures in Exploring Language Texts

When it comes to information content, entropy is a key concept used in the information theory which was originally developed in applied settings for the development of telecommunications and cryptography systems. Entropy refers to the largest amount of information transmitted through a communication channel, representing the average information content and the average uncertainty of a discrete variable [19,21]. So far, entropic measures have been widely used and proved appropriate in the field of quantitative linguistics for various purposes, such as investigating the laws in natural languages [22,23,24,25], linguistic complexity [26,27,28], text types [29,30], keyword extraction [31], and making predictions [32,33]. Theoretically, textual entities with higher entropy values suggests more information content carried by an entity and vice versa [29]. It measures the monotony or stereotypy of a text in quantitative linguistic research [34]. Compared with word frequency to profile the feature of a text, this index considers the global distribution of variation and richness of a linguistic entity, for the reason that word frequency distribution is also an important feature to examine language or language use [35]. In other words, it also provides a measure of how frequently and evenly these words occur or are distributed [36]. 

Typically, textual entities are words. In the present study, we adopted both words and part-of-speech (POS) as textual entities for entropy. As claimed by Murphy [37], syntactic labeling is the operation which chooses which lexical features select the phrasal category. The label indicates the meaning of the structure to the conceptual-intentional system [38]. The processes of syntactic category and syntactic labeling are highly related to core lexico-semantic properties of the lexicon, which reveals the uniqueness of the human computational system [39]. In the setting of interpreting, the diversification of word categories in interpreting texts may be a potential feature distinguishing interpreting type. According to Jia and Liang [4], a striking loss of adjectives was found in CI processing compared to SI, suggesting that a lexical category bias exists across interpreting types. Additionally, some quantitative studies demonstrated that word categories of a given language are meaningful in stylistic analysis [30]. Pan et al. [40] compared original poetry texts with their translated versions from the perspective of POS frequency distribution, and discrepancies were observed among different versions of translated works. Similar results were found between traditional poetry and modern poetry [30]. POS entropy is also investigated to distinguish ‘narrative vs. expository’ text types in both English and Chinese [29].

Since entropy is applied in linguistics to investigate the concentration of the frequencies (in other words, the non-uniformity of the distribution of any entities), the present study also uses the quantitative indicator ‘the repeat rate (RR) (a measure of concentration)’ to digitize the distribution properties of words and word category in different types of interpreting texts. It is assumed that the greater the entropy and the smaller the repeat rate value, the more uniformly the frequencies are distributed (in other words, the more heterogeneous is the text) [34,41].

Therefore, to examine the difference of overall informativeness of outputs between CI and SI and to figure out the word and word category distribution patterns of SI and CI texts, we intend to explore the following three questions:

(1) Could the quantitative indicators of word entropy, POS entropy, and repeat rate of words and word category distinguish CI and SI output texts?
(2) If yes, which of the interpreting modes yields more informative and more heterogeneous output interpreting texts?
(3) What are the implications regarding interpreting processing mechanisms underlying varied informativeness in SI and CI?

In the following sections, we will first introduce the corpus used in the present study and the calculation method of entropy and RR, then present the results of linear mixed-effects models. Discussions on the informativeness between CI and SI will be provided. 

## 2. Materials and Methods

### 2.1. Materials

To verify whether the four indicators of the output texts vary significantly between CI and SI, we built a parallel corpus comprised of transcribed real-world interpretations and the source texts of SI and CI. The CI sub-corpus is made up of English interpretations and source speeches given by Chinese Premiers Wen Jiabao and Li Keqiang at the annual press conference of the National People’s Congress from 2007 to 2018, where the Prime Minister met and answered the questions raised by Chinese and overseas journalists. The SI sub-corpus consisted of 30 English interpretations and the Chinese source keynote speeches delivered by Chinese government leaders Xi Jinping, Wen Jiabao, and Li Keqiang on the international forums including sessions of the UN General Debate, the Davos Forum, the BRICS summit, the Boao Forum for Asia, the G20 Summit, the World Economic Forum, the ASEM Summit and China-ASEAN Business and Investment Summit during the same time period. All of the corresponding interpretations were from the interpreters’ mother tongue (Mandarin Chinese) into their second language (English). 

To avoid the potential bias produced by the text size, the sub-corpora of English interpretations were segmented to be of a similar length, without splitting a complete paragraph. Thus, 17 English files of CI and 17 English files of SI were obtained. Statistical results show no significant difference in the sizes of English files between SI and CI (*t*_(32)_ = 1.258, *p* = 0.218). The Chinese source texts were aligned with English interpretations, and a total of 34 Chinese files of CI and SI were obtained. Table 1 is the overview of the corpus.

The selected source speeches of CI and SI are comparable for several reasons. First, the source speeches of CI and SI were all public speeches addressed on internationally high-level conferences during the same time span. All of the materials selected in our study were of similar formality, language register and delivery rate in the political and economic fields. Second, all of the speeches were delivered by the Chinese government heads and interpreted by expert interpreters from the Department of Translation and Interpretation of China’s Ministry of Foreign Affairs. Third, the source speech of CI and SI are comparable in terms of syntactic complexity. Dependency distance is a well-established measure of syntactic complexity and comprehension difficulty. It is defined as “the number of words intervening between two syntactically related words, or their linear position difference in sentence” [42]. We calculated mean dependency distance of SI and CI source texts respectively, and the results showed that there was no significant difference between mean dependency distance of SI source texts (*M* = 3.67, *SD* = 0.17) and CI source texts (*M* = 3.76, *SD* = 0.18), *t*(32) = −1.409, *p* = 0.17, *d* = 0.51.

### 2.2. Methods

The present study employed word entropy and POS entropy to compare the information content in CI and SI. Shannon’s version of entropy *H* could be computed as follows [43]:

(1) $$H_{i}=-\sum p_{i\;}log_{2}p_{i}$$

in which $P_{i\;}$represents the probability of the *i*-th language unit in the text. 

To calculate word entropy and POS entropy, it is important to reliably approximate the probabilities of word types and POS types first, which can be estimated via the maximum likelihood method [44]. In other words, we need to calculate the relative frequency per word [34] (p. 33):

(2) $$\hat{p}\left(w_{i}\right)=\frac{f_{i}}{\sum_{j=1}^{V}f_{j}^{\prime }}$$

where *f_i_* stands for the token frequency of each word type *W_i_* in a text (or the frequency of each POS types), and V is the total number of types. For example, in the following sentence:

**The** most immediate **and** important goal of our package plan is to reverse **the** economic downturn **and** maintain steady **and** relatively fast growth.

The corresponding word categories of each word are: ‘det’(determiner), ‘adv’(adverb), ‘adj’(adjective), ‘conj’(coordinating conjunction), ‘adj’, ‘noun’, ‘adp’(adposition), ‘pron’(pronoun), ‘noun’, ‘noun’, ‘verb’, ‘part’(particle), ‘verb’, ‘det’, ‘adj’, ‘noun’, ‘conj’, ‘verb’, ‘adj’, ‘conj’, ‘adv’, ‘adj’ and ‘noun’, respectively.

In this example, the word type *the* occurs twice, *and* occurs three times, etc. the overall number of word tokens is 23. Thus, probabilities of

$$\hat{p}\left(the\right)=\frac{2}{23},\;\hat{p}\left(and\right)=\frac{3}{23},\;\mathrm{etc}.$$

Assume a text is a random variable *T* consisting of tokens from a set of word types *V* = {*W_1_, W_2_,..., W_w_*} in which *W* is the theoretical vocabulary size, and the word entropy of T can be calculated as [19]:

(3) $$H\left(T\right)=-\sum_{i=1}^{W}p\left(w_{i}\right)\log_{2}p\left(w_{i}\right)$$

In the above example,

H(T) =−($\frac{2}{23}\log_{2}\left(\frac{2}{23}\right)\;$+ $\frac{3}{23}\log_{2}\left(\frac{3}{23}\right)$ +…+$\frac{1}{23}\log_{2}\left(\frac{1}{23}\right)$) ≈ 4.23 bits/word.

Similarly, for the POS entropy, the category ‘adj’ and ‘noun’ share the highest frequency *f* = 5 and probability *P* = $\frac{5}{23}$, and the category ‘pron’, ‘part’ and ‘adp’ share the lowest value of frequency *f* = 1. Thus, POS entropy *H =* −($\frac{5}{23}\log_{2}\left(\frac{5}{23}\right)\;$+ $\frac{5}{23}\log_{2}\left(\frac{5}{23}\right)$ +…+$\frac{1}{23}\log_{2}\left(\frac{1}{23}\right)$) ≈ 2.93

Another commonly applied indicator of dispersion in the field of linguistics was Repeat Rate, which was introduced by Herdan [45]. It is defined as follows:

(4) $$RR=\frac{1}{N^{2}}\sum_{i=1}^{V}f_{i}^{2}$$

where *N* means the length of the text, and *V* represents the number of word types (or word category types). $f_{i}^{}$ denotes the frequency of the *i*-th word (or word category). Therefore, RR of words in the example sentence equals 0.059, and RR of POS in the example sentence is 0.149.

In the current research, the Chinese source materials were segmented before all texts were put in the Stanford POS Tagger 4.2.0, a software that reads text and assigns the part of speech to each word [46]. Manual check of the tagged results was performed before the computation of entropy and RR. Entropy and RR of SI and CI sub-corpora were computed with the software QUITA [47]. The data is available in the Supplementary Materials.

## 3. Results

We compared the data of SI and CI output texts using two measures with four indicators. Differences of output word entropy, POS entropy, word RR and POS RR were examined by a series of linear mixed-effects models first, and then further analysis was conducted to rule out the potential confounding factor of individual styles of interpreters.

### 3.1. Comparison of Output Word Entropy between SI and CI

**Figure 1:**
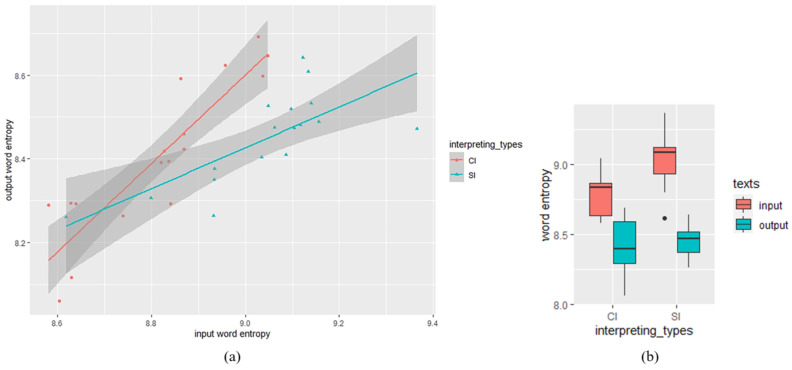
Word entropy analysis: (**a**) scatter plot visualizing the relationship between input and output word entropy in SI and CI (the datapoints indicate word entropy of each text); (**b**) descriptive statistics: input and output word entropy in SI and CI texts.

Descriptive statistics of the word entropy measure showed that the mean value of word entropy in CI outputs was lower (*M* = 8.403, *SD* = 0.183) than SI outputs (*M* = 8.446, *SD* = 0.110). The SI inputs yielded a higher word entropy (*M* = 9.040, *SD* = 0.164) than CI inputs (*M* = 8.813, *SD* = 0.155). Given that interpreting is a process mediating between source language and target language, the difference of output entropy and RR between CI and SI might be caused by the variance of input texts. To figure out the difference of output word entropy between SI and CI, the data was analyzed using linear mixed-effects regressions based on the *lme4* package [48] in R [49], with input word entropy, interpreting types (CI/SI) and their interaction as fixed effects, and interpreters were treated as random effects. The input word entropy was zero-centered before entering analysis, and the predictor of interpreting types was contrasted-coded (SI = −0.5, CI = 0.5). The maximal random-effects structure including the random slopes for interpreters failed to converge, and finally we got the best-fitted model with the random intercepts for interpreters included. As shown in Table 2, our analysis revealed a significant effect of input word entropy (*β* = 0.77, *SE* = 0.091, *t* = 8.43, *df* = 28.67, *p* <0.01). The main effect of interpreting types is also significant (*β* = 0.12, *SE* = 0.035, *t* = 3.4, *df* = 29.3, *p* <0.01), with output word entropy in CI texts significantly higher than that in SI texts. There is a significant interaction of input word entropy and interpreting types (*β* = 0.51, *SE* = 0.18, *t* = 2.8, *df* = 28.58, *p* <0.01), indicating that the output entropy in CI texts is higher than that in SI texts when input word entropy is relatively high. The descriptive statistics and the scatter plot visualizing the relationship between input and output word entropy in SI and CI were exhibited in Figure 1.

### 3.2. Comparison of Output POS Entropy between SI and CI

**Figure 2:**
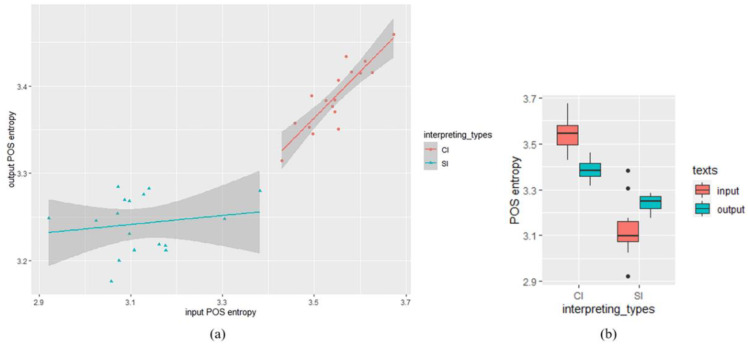
POS entropy analysis: (**a**) scatter plot visualizing the relationship between input and output POS entropy in SI and CI (the datapoints indicate POS entropy of each text); (**b**) descriptive statistics: input and output POS entropy in SI and CI texts.

Descriptive statistics of the POS entropy measure showed that the POS entropy of CI outputs (*M* = 3.388, *SD* = 0.038) was higher than that of SI outputs (*M* = 3.243, *SD* = 0.033). The POS entropy of CI inputs (*M* = 3.546, *SD* = 0.062) was higher than that of SI inputs (*M* = 3.122, *SD* = 0.104). To examine the difference of output POS entropy between SI and CI, another linear mixed-effects analysis was performed, with input POS entropy, interpreting types (CI/SI) and their interaction as fixed effects, and interpreters were treated as random effects. Similarly, the input POS entropy was zero-centered before entering analysis, and the predictor of interpreting types was contrasted-coded (SI = −0.5, CI = 0.5). As shown in Table 3, a significant effect of input POS entropy was found (*β* = 0.31, *SE* = 0.065, *t* = 4.7, *df* = 21.52, *p* < 0.01). No significant main effect was observed in interpreting types (*β* = 0.024, *SE* = 0.028, *t* = 0.84, *df* = 20.34, *p* = 0.41). Critically, we found a significant interaction of input POS entropy and interpreting types (*β* = 0.44, *SE* = 0.13, *t* = 3.35 *df* = 21.15, *p* ≤ 0.01). The descriptive statistics and the scatter plot visualizing the relationship between input and output POS entropy in SI and CI was shown in Figure 2.

### 3.3. Comparison of Output Word RR and POS RR between SI and CI

**Figure 3:**
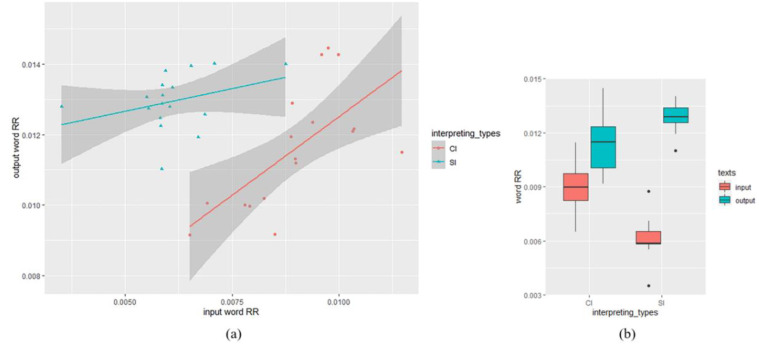
Word RR analysis: (**a**) scatter plot visualizing the relationship between input and output Word RR in SI and CI (the datapoints indicate word RR of each text); (**b**) descriptive statistics: input and output Word RR in SI and CI texts.

As for the difference of output word RR and POS RR between SI and CI, similar methods were adopted. Descriptive statistics showed that word RR of the SI output texts (*M* = 0.013, *SD* = 0.0008) was higher than that of CI output texts (*M* = 0.012, *SD* = 0.0017), while word RR of the SI input texts (*M* = 0.006, *SD* = 0.001) was lower than that of CI input texts (*M* = 0.009, *SD* = 0.0013). The result of the mixed-effects model of word RR was shown in Table 4. There was a significant effect of input word RR (*β* = 0.55, *SE* = 0.17, *t* = 3.22, *df* = 29.84, *p* < 0.01). Our analysis also showed a significant effect of interpreting types (*β* = −0.003, *SE* = 0.0006, *t* = −4.69, *df* = 29.72, *p* < 0.01), with the output word RR in SI texts higher than that in CI texts. No significant interaction was found between input word RR and interpreting types (*β* = 0.57, *SE* = 0.34, *t* = 1.67 *df* = 29.4, *p* = 0.11). Figure 3 shows the relationship between input and output word RR in SI and CI. 

**Figure 4:**
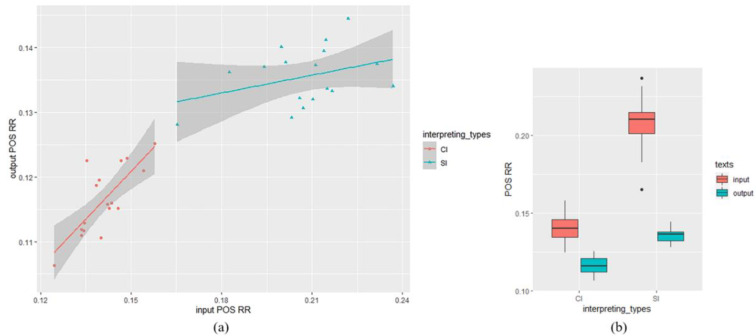
POS RR analysis: (**a**) scatter plot visualizing the relationship between input and output POS RR in SI and CI (the datapoints indicate POS RR of each text); (**b**) descriptive statistics: input and output POS RR in SI and CI texts.

For POS RR, the value of SI output texts (*M* = 0.136, *SD* = 0.004) was higher than CI output texts (*M* = 0.116, *SD* = 0.005), and SI input texts (*M* = 0.208, *SD* = 0.008) yielded a higher value of RR than CI input texts (*M* = 0.141, *SD* = 0.004). The result of the mixed-effects model of POS RR was shown in Table 5. There was a significant of input POS RR (*β* = 0.28, *SE* = 0.051, *t* = 5.43, *df* = 22.71, *p* < 0.01). No significant effect was found in interpreting types (*β* = −0.0035, *SE* = 0.0035, *t* = −1.01, *df* = 24.59, *p* = 0.32), however, our analysis revealed a significant interaction of input POS RR and interpreting types (*β* = 0.43, *SE* = 0.1, *t* = 4.22, *df* = 22.84, <0.001). The descriptive statistics and the scatter plot visualizing the relationship between input and output word RR in SI and CI was shown in Figure 4.

Additionally, since the English interpretations in our CI sub-corpora were interpreted by three different interpreters, the individual styles of interpreters might influence the output indicators’ values [50]. The three interpreters are all highly professional interpreters, working as commissioners of the Translation Department of China’s Ministry of Foreign Affairs. FEI Shengchao performed the CI from 2007 to 2009, ZHANG Lu from 2010 to 2012 and from 2014 to 2018, and SUN Ning in 2013. To rule out this potential factor, we compared output word entropy produced by different interpreters in CI. No significant difference was observed across different interpreting styles (*F* = 2.790, *p* = 0.098, partial η^2^ = 0.3). Thus, the confounding factor of individual interpreting style was ruled out.

## 4. Discussions

The present study is the very first effort of its kind to examine the overall informativeness and concentration of the interpreting texts of CI and SI by employing the entropic measure. Given the difficulty of quantifying complexity in information, previous studies on information transmission in interpreting generally adopted traditional methods such as case studies, experiments, and qualitative analysis, focusing on specific issues of accuracy, information loss, etc. Our study, however, employed a novel method of entropy to scientifically quantify the overall informativeness of texts in interpreting. In this way, different mechanisms of information processing during CI and SI can be explored. Our results indicate that entropy and RR of interpreting outputs can classify interpreting modes, and that there are interactions between input entropy/RR and interpreting types, providing further evidence of the differences between SI and CI in terms of distribution patterns of interpreting texts. Specifically, CI outputs yield a markedly higher value of word entropy than SI outputs, the opposite of the results of word RR. Moreover, when the input entropy/RR values are high, CI outputs show higher degree of complexity in terms of word and word category than that of SI outputs. The statistical analysis reveals that CI outputs are more heterogeneous and informative when input texts have a higher degree of complexity. This suggests that CI interpreters can achieve an equilibrium between production economy for interpreters and comprehension sufficiency for listeners, especially when dealing with more complex input speeches. 

As introduced in the previous section, entropy is a measure of equilibrium or uniformity of language unit frequency distribution, and RR is a measure of dispersion [19,34,41]. The more evenly distributed word frequency in CI suggests that CI is a cognitive process that probably reaches an equilibrium between production economy for interpreters and comprehension sufficiency for listeners, especially in the case where the input speech is more complex. On the one hand, the need for production economy for consecutive interpreters derives from heavy pressure on memory in CI. As postulated by Cowan [51], cognitive load can be measured by the number of chunks held in the focus of attention. In the process of CI, interpreters have to keep more chunks of information in the focus of attention before a long segment of speech is interpreted in one stretch, and the cognitive load may keep accumulating during the course [16]. Facing a high cognitive burden, consecutive interpreters may have an inherent tendency towards using high-frequency words, which conforms to the principle of least effort [52]. 

On the other hand, more even frequency distribution of language units in CI than SI reveals that consecutive interpreters may not rely on common words to describe the source message, which means they may use more concrete words in some expressions to achieve comprehension sufficiency for the audience. Since SI interpreters are more inclined to follow the speakers closely, CI interpreters are more advantaged to mediate between speakers and the audience, thereby introducing more shifts [53]. For speaker’s sake, CI interpreters attempt to reproduce the original message fully and “maximizing information recovery” [10] (p. 211). For target audience’s sake, CI interpreters make efforts to “accommodate target communicative conventions and the demands of target audience” [53] to “maximize the communication impact of the speech” [10] (p. 211). Therefore, the distribution pattern of words in CI outputs is more diverse and fruitful.

In the sense of information theory, entropy is used to quantify linguistic features in terms of diversity or complexity [54,55,56], and the entropy of words can be seen as the upper bound on expressivity [22]. This indicator is drawn on to measure the freedom of choice, that is the uncertainty on choosing word categories and words. Our findings signify that CI outputs are more diverse and complicated, and the use of words are more unpredictable as compared to SI outputs, which conforms to the explicitation hypothesis. ‘Explicitation’ was initiated as ‘making explicit in the target language what remains implicit in the source language since it is apparent from either the context or the situation’ [57]. In CI, the comprehension phase and the reformulation phase are separated. The reformulation phase is not started until the input speech in the source language is fully processed. This enables CI interpreters to have longer time to integrate the message of several sentences in the source language and form an overall understanding of the core point as well as the context. With a macro structure of the source text in mind, CI interpreters can make the renderings more explicit by providing additional information which can be inferred from the context. From the interpreter’s perspective, the motivation of using explicitness in CI may be to fill in the gap arising from information loss. To avoid causing a ‘vacuum of meaning’ for listeners, consecutive interpreters resort to the strategy of explicitation and fill in these gaps with explicit information that is inferable from other contextual information [58]. From the listener’s perspective, consecutive interpreters have more time for clarifying and explaining the original information to achieve comprehension sufficiency.

Additionally, the more informative CI outputs is consistent with the deverbalization hypothesis, a strategy that interpreter generally captures the speaker’s intended sense rather than relying on a linguistic conversion of words and phrases [59]. It has been demonstrated in previous research that memory for the verbatim surface forms lasts for only a few seconds [60]. Since the utterances of the target texts in CI are produced with a time lag after the corresponding source speech, consecutive interpreters are inclined to discard the linguistic form of the source language to relieve memory pressure [61]. Consequently, it is not possible for consecutive interpreters to interpret in the word-for-word consistency between the source and target speech, which may result in a higher degree of uncertainty in CI outputs than SI outputs.

Conversely, simultaneous interpreters are constrained in the amount of time available and the linear processing behavior. Firstly, the simultaneity of comprehension and production in SI makes it hard for simultaneous interpreters to get the gist and context of the source message. SI is instantaneously produced at the time when simultaneous interpreters are exposed to the source speech, and then continuous input of new information needs to be processed. Due to the limited capacity of working memory, the information awaiting processing can only be stored for several seconds. Under high time pressure, interpreters have to process very fast to keep up with the flow of information. In this regard, word choice would be relatively monotonous for simultaneous interpreters, and it would be easier for SI outputs to concentrate on a particular word. 

According to the principle of ‘uniform information density’ [62,63], language users make choices that keep the amount of information communicated per unit of time approximately constant. For instance, less informative syllables are produced with shorter durations than more informative syllables. In other words, the informativeness conveyed by a word is linearly related to the amount of time it takes to produce [24]. Unlike CI where interpreters have an adequate period of time to reformulate the target speech, SI is challenging in terms of the processing time. Therefore, the limited time for delivering target speech renders the SI outputs less informative than CI outputs. 

Secondly, in SI, where structurally and syntactically different languages are involved, the linear processing behavior could interfere the interpreter’s freedom in word choice. It is claimed that the direct interpretation of short units demands less cognitive resources [13]. To minimize the effort for attention switching and information construction, simultaneous interpreters generally tend to perform strategically, by following the source texts in a linear manner to achieve the maximum communicative capacity [13]. In this case, the form of the source text is retained to the largest extent in the processing of SI, and the choice of word in SI outputs are less heterogeneous but more predictable compared with CI outputs. 

Critically, our findings about the interaction of input entropy/RR and interpreting types shed lights upon application scenarios of interpreting. The complexity of input speeches in terms of the frequency distribution of word/ word category can be one of the criteria in selecting interpreting types. For more complex input speeches, CI may be more advantageous in improving interpreting efficiency. As discussed above, CI interpreters comprehend the input speeches based on a complete paragraph of message due to a longer time lag before producing the outputs. With more context information in mind, they can comprehend complex input speeches more easily. Meanwhile, the time lag gives CI interpreters more time to reconstruct the output sentences in a clearer manner. However, facing complex inputs, SI interpreters may miss some points of information. Such a loss may be triggered by the interference of complex input speech structures and heavier cognitive load regarding the simultaneity of listening and speaking. Conversely, for relatively simple input speeches, SI may be a better choice than CI, for the reason that simple inputs enable interpreters to keep more details of the original information. In this case, SI interpreters almost do not need to deal with complicated words and sentence structures. Simple vocabulary can be easily retrieved from interpreters’ long-term memory, and each chunk of the source message can be interpreted promptly, which helps retain the details to the largest extent. Compared to SI, facing less complex source speeches, CI interpreters are unable to align every detail to the source message. Therefore, SI may be more advantageous in the situation where the input speeches are less complex. 

Our investigation into the distinct features of interpreted texts between SI and CI in terms of informativeness also serves as a mirror for language foundations. Interpreting is a complex verbal activity where cognitive processing and language use interact [64]. More informative outputs observed in CI can be regarded as an adaptive behavior of language to mediate between cognitive pressure and information expression. This suggests “the need for languages to offer efficient communication between humans given our cognitive constraints” [65]. Similar to the findings in the grammar and tense systems across languages [65,66], language in the setting of interpreting seems to find a balance between production economy for the speaker and the comprehension sufficiency for the hearer. Also, our results are in line with the claim that “languages are well-designed for human use” [67,68] in the context of interpreting tasks. As proposed by Gong and Shuai [69], there is a coevolution scenario between language and memory capacity, and the limited memory capacity drives language transition. The discrepancy of informativeness between CI outputs and SI outputs illustrates how language shifts under great cognitive load and provides evidence for the view that language is a system with the feature of ‘self-organization’ in terms of distribution of linguistic units [43].

The current research not only complements previous strands of research on the distinction among interpreting types and taps into a new perspective of information processing between SI and CI, but also underscores that language is shaped by cognitive constraints and socio-cultural environment. Together with other quantitative studies on interpreting types, our study complements research on fully-fledged common language uses and offers a new perspective for the understanding of language evolution.

## 5. Conclusions

The present study adopted a novel entropy-based approach to compare the informativeness of interpreted texts between SI and CI. Our results show that entropy and RR can distinguish CI and SI outputs and that CI outputs entail a higher word entropy value and a lower word RR than SI outputs. We propose that CI is a cognitive process which reaches an equilibrium between production economy for interpreters and comprehension sufficiency for listeners, especially in the case where input speeches are more complex. CI is more advantageous for complex input speeches, lending support to the explicitation and deverbalization hypotheses in CI. Moving forward from the theoretical differences to application scenarios, our findings also shed lights upon the selection of interpreting types in different situation. Furthermore, the current research demonstrates a dynamic adaptation of language users to extreme cognitive load. 

Future avenues of research might dig deeper into more local features of interpreted texts in terms of informativeness at the sentence-position level. The entropic measure can be integrated with experimental research or other quantitative methods to provide further evidence on this topic. Finally, more language pairs should be further examined to explore universals in the interpreting processing in terms of the information flow.

## Figures and Tables

**Table 1:** Overview of the Corpus.

Sub-Corpora	Chinese/English	Text Count	Overall Size
CI	English	17	86,529
	Chinese	17	65,590
SI	English	17	86,017
	Chinese	17	63,678

**Table 2:** Fixed effects in the linear mixed effects model of word entropy.

	Predictor	Estimate	*SE*	*t*-Value	*p*
Output word entropy	Intercept	8.44	0.022	383.56	<0.001 *
	Input word entropy	0.77	0.091	8.43	<0.001 *
	Interpreting types	0.12	0.035	3.40	0.002 *
	Input word entropy × Interpreting types	0.51	0.18	2.80	0.009 *

* *p* < 0.05; SE = standard error.

**Table 3:** Fixed effects in the linear mixed effects model of POS entropy.

	Predictor	Estimate	*SE*	*t*-Value	*p*
Output POS entropy	Intercept	3.28	0.016	202.83	<0.001 *
	Input POS entropy	0.31	0.065	4.70	<0.001 *
	Interpreting types	0.024	0.028	0.84	0.41
	Input POS entropy × Interpreting types	0.44	0.13	3.35	0.003 *

* *p* < 0.05; SE = standard error.

**Table 4:** Fixed effects in the linear mixed effects model of word RR.

	Predictor	Estimate	*SE*	*t*-Value	*p*
Output word RR	Intercept	0.01	0.00033	35.813	<0.001 *
	Input POS entropy	0.55	0.17	3.22	0.0031 *
	Interpreting types	−0.0029	0.00061	−4.69	<0.001 *
	Input POS entropy × Interpreting types	0.57	0.34	1.67	0.11

* *p* < 0.05; SE = standard error.

**Table 5:** Fixed effects in the linear mixed effects model of POS RR.

	Predictor	Estimate	*SE*	*t*-Value	*p*
Output POS RR	Intercept	0.13	0.0019	70.42	<0.001 *
	Input POS entropy	0.28	0.051	5.43	<0.001 *
	Interpreting types	−0.0035	0.0035	−1.01	0.32
	Input POS entropy × Interpreting types	0.43	0.1	4.22	<0.001 *

* *p* < 0.05; SE = standard error.

## Data Availability

The data presented in this study are available in the Supplementary Materials.

## Supplementary Materials

The following supporting information can be downloaded at: https://www.mdpi.com/article/10.3390/e25020243/s1, Table S1: Word entropy and POS entropy of CI and SI texts; Table S2: Word RR and POS RR of CI and SI texts.
