# Supplementary material for: Informativeness across Interpreting Types: Implications for Language Shifts under Cognitive Load
Source: Entropy (Basel). 2023 Jan 28;25(2):243. doi: 10.3390/e25020243 (PMC9955845; doi:10.3390/e25020243)
Supplement: Supplementary file 1 [file entropy-25-00243-s001.zip › entropy-2095631-supplementary.pdf]

Supplementary Materials

Table S1. Word entropy and POS entropy of CI and SI texts.

Text Number	Word entropy				POS entropy			
	CI output	SI output	CI input	SI input	CI output	SI output	CI input	SI input
1	8.292	8.475	8.638	9.062	3.384	3.246	3.545	3.025
2	8.293	8.261	8.629	8.618	3.459	3.218	3.673	3.162
3	8.116	8.376	8.63	8.935	3.415	3.283	3.626	3.14
4	8.06	8.489	8.604	9.156	3.434	3.268	3.569	3.097
5	8.419	8.532	8.827	9.14	3.416	3.276	3.58	3.128
6	8.289	8.472	8.581	9.366	3.415	3.249	3.601	2.921
7	8.263	8.526	8.739	9.049	3.407	3.231	3.552	3.097
8	8.395	8.306	8.836	8.799	3.428	3.176	3.61	3.057
9	8.292	8.608	8.841	9.134	3.35	3.212	3.553	3.108
10	8.423	8.481	8.87	9.117	3.39	3.212	3.494	3.177
11	8.598	8.519	9.037	9.097	3.353	3.248	3.49	3.304
12	8.592	8.642	8.863	9.122	3.358	3.217	3.458	3.176
13	8.391	8.35	8.821	8.933	3.383	3.2	3.525	3.074
14	8.459	8.409	8.869	9.087	3.37	3.284	3.545	3.072
15	8.623	8.404	8.957	9.035	3.377	3.27	3.54	3.087
16	8.647	8.263	9.048	8.932	3.315	3.28	3.43	3.382
17	8.692	8.474	9.027	9.104	3.345	3.253	3.496	3.071

Table S2. Word RR and POS RR of CI and SI texts.

Text Number	Word RR				POS RR			
	CI output	SI output	CI input	SI input	CI output	SI output	CI input	SI input
1	1.22%	1.34%	1.03%	0.59%	11.51%	13.75%	14.27%	23.16%
2	1.21%	1.40%	1.03%	0.88%	10.63%	13.70%	12.45%	19.41%
3	1.45%	1.26%	0.97%	0.69%	11.09%	12.91%	13.36%	20.33%
4	1.43%	1.27%	1.00%	0.56%	11.06%	13.20%	14.00%	21.03%
5	1.23%	1.23%	0.94%	0.58%	11.17%	13.21%	13.43%	20.59%
6	1.15%	1.28%	1.15%	0.35%	11.19%	13.40%	13.36%	23.69%
7	1.29%	1.31%	0.89%	0.59%	11.58%	13.95%	14.22%	21.40%
8	1.19%	1.40%	0.89%	0.71%	11.29%	14.45%	13.45%	22.20%
9	1.43%	1.29%	0.96%	0.59%	12.25%	14.12%	13.54%	21.47%
10	1.13%	1.33%	0.90%	0.61%	11.51%	13.77%	14.57%	20.13%
11	1.00%	1.19%	0.78%	0.67%	12.29%	13.62%	14.86%	18.26%
12	0.92%	1.10%	0.85%	0.59%	12.09%	14.01%	15.40%	19.99%
13	1.12%	1.38%	0.90%	0.59%	11.59%	13.73%	14.35%	21.12%
14	1.02%	1.25%	0.82%	0.58%	11.87%	13.06%	13.85%	20.70%
15	1.00%	1.28%	0.79%	0.60%	11.95%	13.33%	13.93%	21.67%
16	1.00%	1.39%	0.69%	0.65%	12.52%	12.81%	15.79%	16.53%
17	0.92%	1.31%	0.65%	0.55%	12.25%	13.36%	14.67%	21.50%
